# Inequalities in anemia among Peruvian children aged 6–59 months: A decomposition analysis

**DOI:** 10.3389/fpubh.2023.1068083

**Published:** 2023-03-31

**Authors:** Ali Al-kassab-Córdova, Carolina Mendez-Guerra, Pamela Robles-Valcarcel, Luis Iberico-Bellomo, Kenedy Alva, Percy Herrera-Añazco, Vicente A. Benites-Zapata

**Affiliations:** ^1^Centro de Excelencia en Investigaciones Económicas y Sociales en Salud, Universidad San Ignacio de Loyola, Lima, Peru; ^2^Facultad de Ciencias de la Salud, Universidad Peruana de Ciencias Aplicadas, Lima, Peru; ^3^Facultad de Economía y Finanzas, Universidad del Pacifico, Lima, Peru; ^4^Escuela de Negocios y Administración de Empresas, Universidad de Murcia, Murcia, España; ^5^Facultad de Ciencias de la Salud, Universidad Privada del Norte, Trujillo, Peru; ^6^Red Peruana de Salud Colectiva, Lima, Peru; ^7^Maestría de Epidemiología Clínica y Bioestadística, Universidad Científica del Sur, Lima, Peru

**Keywords:** anemia, children, healthcare inequalities, demographic and health survey, Peru

## Abstract

**Objective:**

To quantify the inequalities of anemia in Peruvian children aged 6–59 months and uncover its contributing factors.

**Materials and methods:**

We conducted a cross-sectional study based on the secondary data analysis of the 2021 Peruvian Demographic and Health Survey (DHS). Our sample included Peruvian children aged 6–59 months with complete data for the variables of interest. Anemia was defined as having a hemoglobin level of less than 11 g/dL, adjusted by altitude. Erreygers Concentration Index (ECI) and concentration curves were computed to estimate the socio-economic inequality in anemia among Peruvian children. Moreover, ECI was decomposed to figure out the contributing factors to the inequality of anemia and the residual variation.

**Results:**

Nationwide, the prevalence of anemia in Peruvian children was 29.47%. We found a pro-poor inequality regarding anemia at the national level (ECI = −0.1848). The determinants included in the model explained 81.85% of the overall socio-economic inequality in anemia. The largest contribution to inequality was from household- and community-related factors. Having a higher mother’s education level (26.26%) and being from the highlands (24.91%) were the major significant contributors to the overall health inequality.

**Conclusion:**

Almost one-third of Peruvian children have anemia. A pro-poor inequality of anemia in Peruvian children was found. Public policies ought to address the major contributing factors of anemia inequality.

## 1. Introduction

Anemia is a highly prevalent and inequitable medical condition among low- and middle-income countries, especially in the Latin American and Caribbean (LAC) region (1, 2). Although its severity has been declining in recent years, it is still the leading cause of Years Lived with Disability among children and adolescents (3), and a risk factor for children’s mortality (4). But its scope goes beyond the acute affection of children as it produces deleterious effects during adolescence (such as more vulnerability to infection, impaired physical growth and performance, and reduced mental development and school achievements) and adulthood (such as low productivity, impaired quality of life and diminished general income) (5–7). As a result, it is translated into economic-related consequences on human capital, and in turn, affects the economy of the country.

Unfortunately, Peru has one of the highest prevalence of childhood anemia in the LAC region (8), which poses a latent public health problem and a continued threat to the national economy. Nationwide, anemia generates a major economic burden as its costs ascend to 0.62% of the gross domestic product, mostly related to cognitive impairment in adulthood. In this regard, 46.3% of the cost of anemia to the Peruvian State is due to cognitive loss, 12.7% to loss of schooling, and 18.2% to loss of productivity in adults (9). Thereby, anemia still jeopardizes the future of the economy owing to its long-term consequences on human resources.

Although childhood anemia has been associated with socioeconomic status (SES) in several countries from the LAC region (10), including Peru (11), few studies have quantified the magnitude of inequality of anemia in Peru and none have figured out its contributors (1, 12). A previous study on Peruvian children unveiled high-risk clusters of anemia throughout the national territory. In fact, there are geographic variations according to area and region of residence, suggesting the presence of regional inequalities (13), which might be determined by modifiable factors. Indeed, certain sociodemographic and nutritional factors have been associated with anemia in children (11). Hence, our study sought to quantify the inequalities of anemia in Peruvian children aged 6–59 months and uncover its contributing factors.

## 2. Materials and methods

### 2.1. Study design, data sources, and selection criteria

We performed a cross-sectional study based on the secondary data analysis of the 2021 Peruvian Demographic and Health Survey (DHS), which is collected by the National Institute of Statistics and Informatics (INEI, from the Spanish acronym). Peru is an upper-middle-income country located in South America. It is administratively divided into departments, provinces, and districts. The Peruvian DHS is annually conducted and has national, regional, and area of residence representativeness. The survey had a probabilistic, stratified, two-stage, independent, and self-weighted design. It included 36,760 households in 2021. Further details regarding the Peruvian DHS are described elsewhere (14). Children aged 6–59 months with complete data for the variables of interest were included in the analysis.

### 2.2. Outcome definition

Anemia is a dummy variable defined, according to the World Health Organization (WHO), as having a hemoglobin level of less than 11 g/dL. Additionally, to define severity it was categorized as mild (10–10.9 g/dL), moderate (7.0–9.9 g/dL), and severe (<7 g/dL) (15). Hemoglobin concentration was measured using a HemoCue® Hb201 portable hemoglobinometer. After, it was adjusted by altitude using the formula designed by the US Centers for Disease Control and Prevention (16).

### 2.3. Explanatory variables

**Figure 1:**
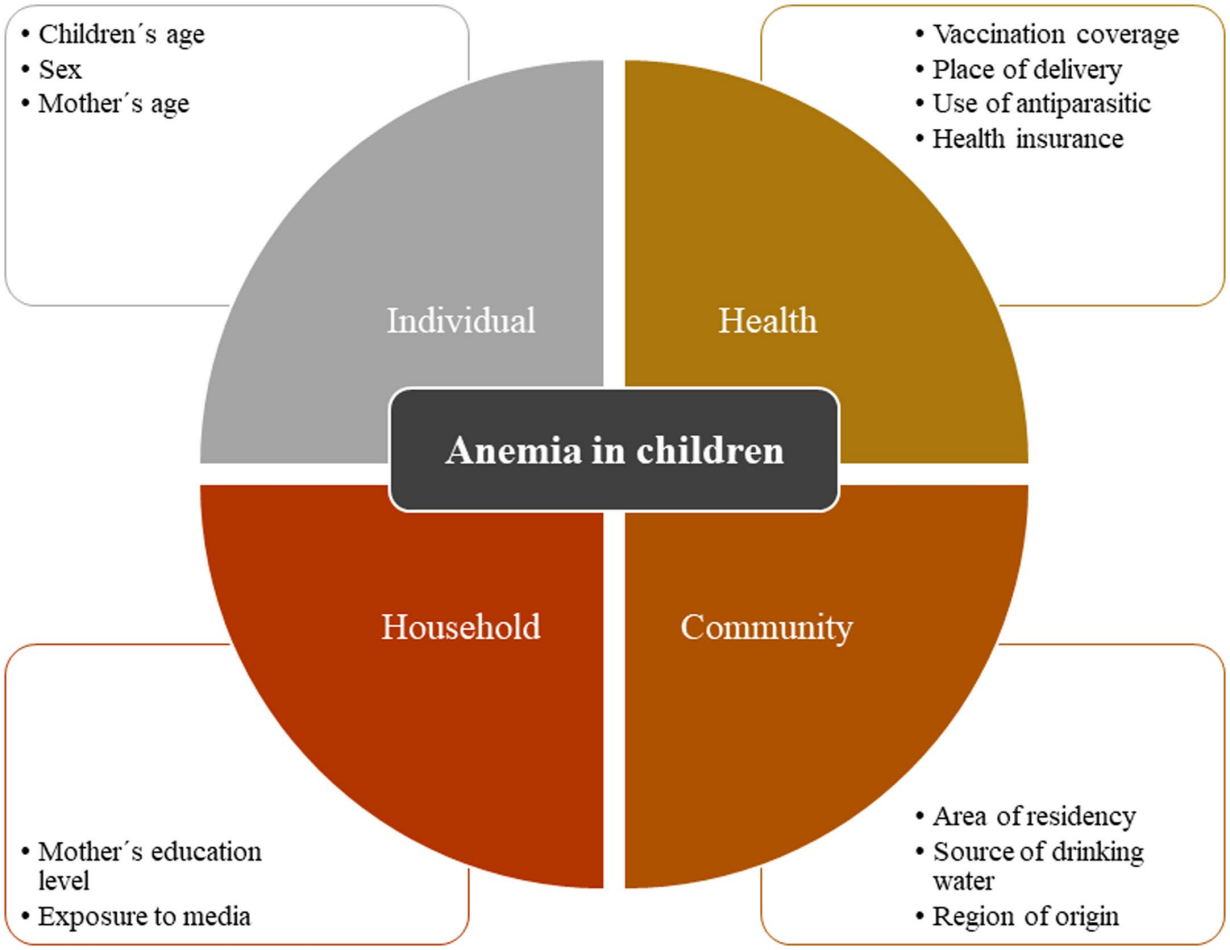
Framework of anemia in children ^*^Adaptation based on the framework by Ngnie-Teta et al. (17).

The selection of the potential explanatory variables was based on the framework designed by Ngnie-Teta et al., and they were grouped into four groups: individual characteristics, health-related variables, household-related variables, and community-related variables (17). An attempt was made to include the framework baseline variables but, in their absence, proxies were employed. Regarding individual characteristics, we included children’s age (6–23, 24–42, and 43–59 months), sex (male, female), and mother’s age (<25, 25–34, and ≥35 years). The health-related variables were vaccination coverage determined by the WHO definition (complete or incomplete) (18), place of delivery (home, healthcare facility), use of antiparasitic in the last 6 months (yes, no), and health insurance (yes, no). Regarding household-related variables, we included the mother’s education level (no education/primary, secondary, and higher), and exposure to media (yes, no), which included those households that owned internet, radio, or TV. Finally, the community-related variables included were the area of residency (urban, rural), source of drinking water (safe, unsafe), and region of origin (Lima Metropolitan area, rest of the coast, highlands, and jungle; Figure 1) Additionally, the following variables were included only for descriptive purposes: birth weight [low (<2,500 g), normal (2,500–4,000 g), and macrosomia (>4,000 g)], acute malnutrition (not malnourished, moderate, and severe), and exclusive breastfeeding [no (<6 months), yes (≥6 months)].

### 2.4. Socioeconomic status

The wealth index was used as a proxy measure of SES. It was calculated through principal component analysis. To construct this variable, some questions were asked to the participants regarding their household building materials, sanitation and water access, and ownership of several assets. After, individuals were ranked based on the score of the households they live in. Finally, such ranked positions were used to classify individuals into five groups from the poorest to the richest (19).

### 2.5. Statistical analysis

The database was downloaded from the “*Microdatos”* webpage of the INEI (20) and was imported into Stata 16.0 (Stata Corporation, College Station, Texas, EE. UU.) for further analysis. We employed the *svy* package for complex design surveys and we adjusted weights, clusters, and strata. Categorical variables were described as weighted proportions. As well, for inequality analysis, we employed the *Lorenz* and c*onindex* packages (21, 22).

#### 2.5.1. Concentration curve and Erreygers concentration index

Concentration curves (CC) were calculated to estimate the socio-economic inequality in anemia among Peruvian children. The concentration index (CI) reckons inequalities by measuring the area under the curve and is calculated as twice the weighted covariance between the outcome and fractional rank in the wealth distribution divided by the mean of the variable. CI can be represented as follows:


$$C=\frac{2}{\mu }\mathrm{cov}\left(y_{i},R_{i}\right)$$

where C is the CI; $y_{i}$ is the outcome variable index; $R_{i}$ is the fractional rank of individuals in the distribution of socioeconomic position; *μ* is the mean of the outcome variable of the sample; and cov denotes the covariance. Values of the CI close to zero represent the existence of small inequality, while those values of the CI close to ±1 show the existence of greater inequality (23).

Regarding the CC, it represents the distribution of a health variable (anemia) among the cumulative proportions of a specific population classified based on their socioeconomic level: from the poorest to the richest. On the *X*-axis, the distribution of children surveyed is ordered by socioeconomic level, and on its *Y*-axis the proportion of anemia is ordered. If this proportion would be equally distributed among the population based on their income, a 45° diagonal would be generated, and the CI would be equal to zero. A deviation of the curve to either side demonstrates the presence of inequality. A positive value of the CI (curve below the diagonal) means that inequality in access to health is pro-rich, while a negative value of the CI (curve above the diagonal) implies that the inequality is pro-poor. The greater the area under the curve (represented by CI), the greater the inequality (24).

Nevertheless, as the CI depends on a mean value of the health variable (anemia), Wagstaff stated that the CI has a constraint, which occurs when the variable under analysis is binary in nature; since as the mean increases, the CI shrinks (25). As well, Erreygers stated that CI can be applied only to unbounded variables with ratio- or fixed scale (26). Accordingly, Erreygers proposed a correction named Erreygers concentration index (ECI), in order to provide a solution for the drawback of the typical CI, which is more compatible with binary variables. It satisfies the properties of transfer, level independence, cardinal invariance, and mirror (27). It is defined as follows:


$$ECI=\frac{8}{n^{2}\left(b_{h}-a_{h}\right)}\overset{n}{\underset{i=1}{\sum }}z_{i}h_{i}$$

#### 2.5.2. Decomposition of the Erreygers concentration index

To determine the factors that explain the pro-poor inequality observed in childhood anemia and the residual variation, which is not explained by any of the factors analyzed, we performed the decomposition of the ECI. In other words, ECI decomposition is divided into a deterministic and a residual component. This decomposition technique provides an in-depth understanding of the inequality, as it estimates the specific contributions of each potential explanatory factor to income-related health inequality. However, this technique assumes linearity between the variable of interest and its determinants (27). Thus, to fit the decomposition technique, all variables were coded as dummies in the model. We considered a confidence level of 95% to test the significance of the marginal effects from our explanatory variables, derived from a multivariable Probit regression model. ECI decomposition is mathematically depicted as follows:


$$\mathrm{ECI}=4\left[\underset{k}{\sum }\beta_{k}{\bar{x}}_{k}C_{k}+GC_{\in }\right]$$

where $\beta_{k}$ depicts the coefficient of the explanatory variables; ${\bar{x}}_{k}$ is the mean of the explanatory variable; $C_{k}$ denotes the concentration index of the explanatory variables; and $GC_{\in }$ is a generalized concentration for the error term.

According to Erreygers and Kessels (28), it is not recommendable to include SES (*id est*. wealth index in our study) as an independent factor in the regression-based decomposition. If included, the residual component would be close to zero and itself would explain most of the inequality. They state that it would be unnatural to explain the correlation between health and SES by including either of these variables in the decomposition. Due to the above, we did not include SES.

Finally, to assess the robustness of the decomposition technique, we conducted a sensitivity analysis by changing the reference groups (29).

## 3. Results

### 3.1. Descriptive statistics

A total of 18,846 children were included in the analysis (see flowchart in Supplementary Figure 1). About one-third of children were between 6 and 23 months of age (34.08%) and most of them were males (50.54%). Regarding the area of residence, 25.9% of children lived in rural areas and 29.55% had an unsafe source of drinking water. The majority were from the rest of the coast (28.29%). Most mothers were between 24 and 35 years of age (47.71%) and 18.32% of them had no education or primary school. Also, 32.09% of children used antiparasitic in the previous 6 months, 5.91% were born in a non-institutionalized place, and 14.8% did not have health insurance. Likewise, the mean birth weight of the participants was 3456.43 (SD: 1210.36) g and most of them received exclusive breastfeeding and were not malnourished (Table 1).

**Table 1:** Demographic and socioeconomic characteristics of Peruvian children aged 6–59 months.

Baseline characteristics		*n* (%)^*^
Age (months)		
	6–23	6,839 (34.08)
	24–42	7,048 (34.33)
	43–59	6,325 (31.59)
Sex		
	Male	10,219 (50.54)
	Female	9,993 (49.46)
Wealth index		
	Poorest	6,052 (25.09)
	Poor	5,312 (23.45)
	Middle	5,312 (20.6)
	Rich	2,973 (17.49)
	Richest	1,925 (13.37)
Area of residence		
	Urban	13,841 (74.1)
	Rural	6,371 (25.9)
Mother’s age (years)		
	15–24	4,257 (20.67)
	25–34	9,678 (47.71)
	≥ 35 years	6,277 (31.62)
Vaccination coverage		
	Complete	13,017 (64.12)
	Incomplete	7,190 (35.88)
Use of antiparasitic in the previous 6 months		
	Yes	7,186 (32.09)
	No	13,026 (67.91)
Place of delivery		
	Institutional	18,805 (94.09)
	Non-institutional	1,117 (5.91)
Health insurance		
	Yes	17,561 (85.2)
	No	2,651 (14.8)
Source of drinking water		
	Safe	13,445 (70.45)
	Unsafe	6,767 (29.55)
Mother’s education		
	No education or primary	3,869 (18.32)
	Secondary	9,623 (46.63)
	Higher	6,720 (35.05)
Exposure to media (internet, TV, radio)		
	Yes	18,029 (90.72)
	No	2,183 (9.28)
Region of origin		
	Lima (Metropolitan area)	2,522 (26.21)
	Rest of the Coast	5,974 (28.29)
	Highlands	6,650 (27.64)
	Jungle	5,066 (17.87)
Birth weight		
	Mean in grams (SD)	3456.43 (SD: 1210.36)
	Low	1,244 (6.12)
	Normal	17,163 (85.32)
	Macrosomia	1,805 (8.56)
Acute malnutrition		
	Not malnourished	20,133 (99.65)
	Moderate	63 (0.28)
	Severe	16 (0.07)
Exclusive breastfeeding		
	Yes	19,331 (95.26)
	No	881 (4.74)

SD, standard deviation. ^*^All proportions were weighted.

**Figure 2:**
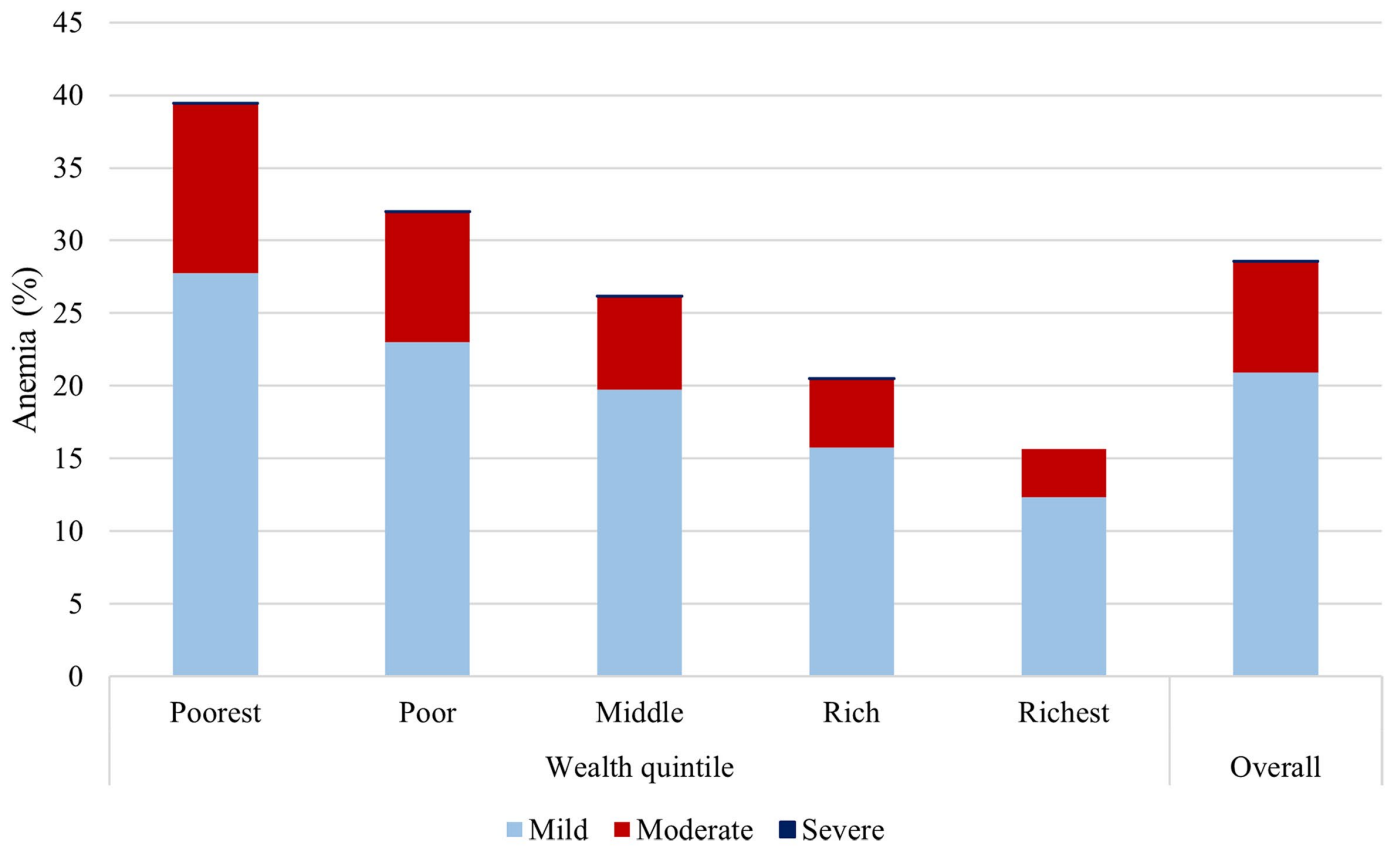
Severity of anemia according to wealth index in Peruvian children.

Nationwide, the prevalence of anemia was 29.47%, of which 20.89% had mild anemia, 7.65% moderate anemia, and less than 1% severe anemia. However, when stratified by wealth index, the poorest children had 39.41% of anemia, whereas the richest children had 15.65%. Moreover, the prevalence of moderate anemia was 11.63% among the poorest, while it was 3.31% among the richest (Figure 2).

### 3.2. Concentration curve and Erreygers concentration index

**Figure 3:**
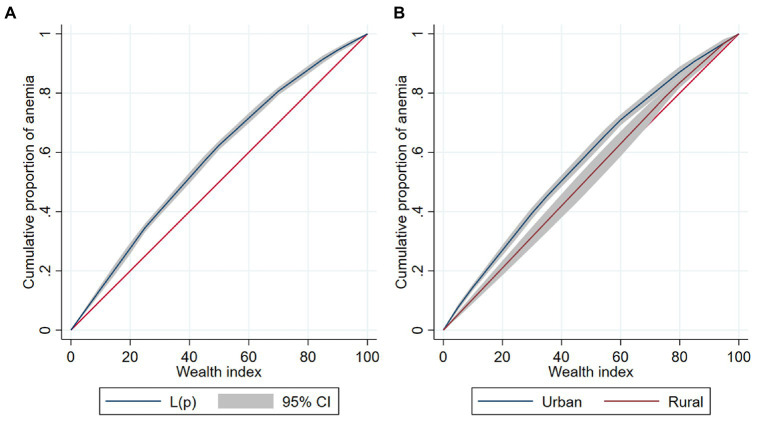
Concentration curve of anemia among Peruvian children **(A)**. Overall concentration curve of anemia. **(B)** Concentration curve of anemia stratified by area of residence.

**Figure 4:**
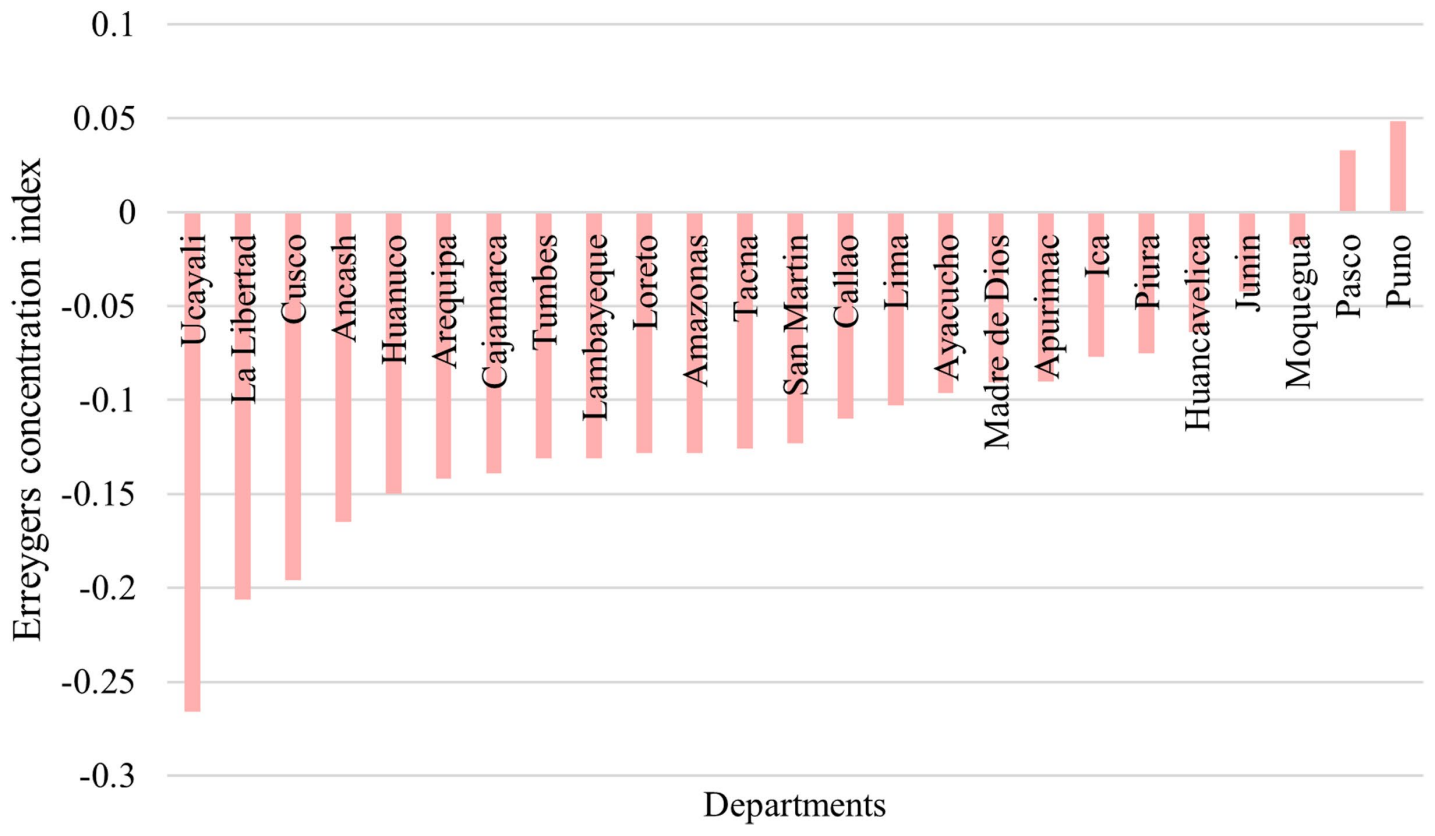
ECI for anemia among Peruvian children by departments.

Anemia among Peruvian children had a pro-poor inequality at the national level (ECI = −0.1848) (Figure 3A). When stratified by area of residence, inequalities were of higher magnitude in urban areas (ECI = −0.1493), compared to rural areas (ECI = −0.0603; Figure 3B). The ECI ranged between −0.2658 and 0.0485 among departments. In all departments, the line was above the equality line (except for Pasco and Puno). The departments with the lowest ECI were Ucayali (ECI = −0.2658), La Libertad (ECI = −0.2063), and Cusco (ECI = −0.1960). Meanwhile, the departments with the highest ECI were Puno (ECI = 0.0485), Pasco (ECI = 0.0328), and Moquegua (ECI = −0.0173; Figure 4; Supplementary Figure 2).

### 3.3. Decomposition of the Erreygers concentration index

**Figure 5:**
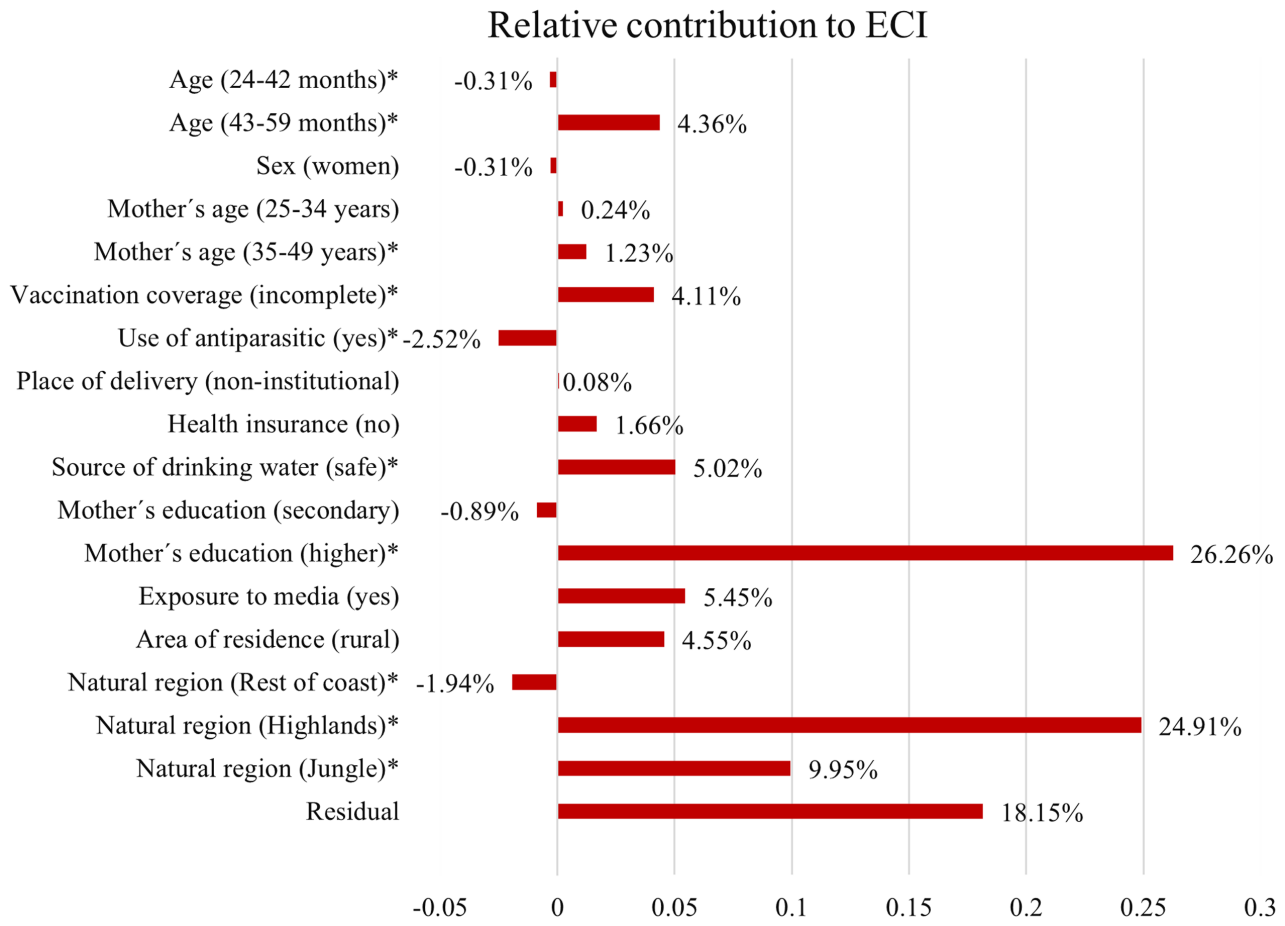
Decomposition of ECI: the relative contribution of each factor to inequality in anemia among Peruvian children. ^*^indicates statistically significant (*p* < 0.05) marginal effect derived from multivariable Probit regression model.

The determinants included in the model explained 81.85% of the total variance of overall socio-economic inequality in anemia among Peruvian children. The largest contribution came from household- and community-related factors. Having a higher mother’s education level (26.26%) and being from the highlands (24.91%) were the major significant contributors to the overall health inequality. As well, children aged between 43 and 59 months (4.36%), mothers aged between 35 and 49 years (1.23%), incomplete vaccination coverage (4.11%), and safe source of drinking water (5.02%) had relatively minimal contribution toward the inequality. Conversely, the use of antiparasitic drugs in the last 6 months negatively contributed to inequality by −2.52%. The contribution due to the residuals (unexplained part) was 18.15% (Table 2; Figure 5).

**Table 2:** Detailed decomposition of Erreygers normalized concentration index.

Contributing factors		Marg. Effects	Elas.	C_k_	% Contr.
*Individual characteristics*					
Children’s age (ref: 6–23 months)					
	24–42 months	−0.779^*^	−0.001	0.001	−0.31
	43–59 months	−1.150^*^	0.007	−0.008	4.36
Sex (ref: Men)					
	Women	0.397^*^	0.001	0.001	−0.31
Mother’s age (ref: 15–24)					
	25–34 years	−0.054	0.008	−0.000	0.24
	≥ 35 years	−0.088^*^	0.0256	−0.002	1.23
*Health-related variables*					
Vaccination coverage (ref: Complete)					
	Incomplete	−0.613^*^	0.012	−0.008	4.11
Place of delivery (ref: Institutional)					
	Non-Institutional	0.003	−0.039	−0.000	0.08
Use of antiparasitic in the last 6 months (ref: No)					
	Yes	−0.117^*^	−0.039	0.005	−2.52
Health insurance (ref: Yes)					
	No	0.134	−0.022	−0.003	1.66
*Household-related variables*					
Mother’s education level (ref: No education/primary)					
	Secondary	−0.043	−0.037	0.002	−0.89
	Higher	−0.384^*^	0.126	−0.048	26.26
Exposure to media (ref: No)					
	Yes	−0.189	0.053	−0.010	5.45
Source of drinking water (ref: Unsafe)					
	Safe	0.137^*^	−0.067	−0.009	5.02
*Community-related variables*					
Area of residency (ref: Urban)					
	Rural	0.052	−0.160	−0.008	4.55
Region of origin (ref: Lima)					
	Rest of the Coast	0.105^*^	0.033	0.003	−1.94
	Highlands	0.568^*^	−0.081	−0.046	24.91
	Jungle	0.295^*^	−0.062	−0.018	9.95
*Sum of ECI*				−0.148	81.85
*Residual (unexplained)*				−0.037	18.15
*Erreygers-corrected concentration index*				−0.184	

^*^indicates statistically significant (*p* < 0.05) marginal effect derived from multivariable probit regression model.

Elas., elasticity (β_k_^*^ x̅_k_ /μ); ECI, Erreygers´ concentration index; and C_k_, Erreygers-corrected concentration index of determinant *k*.

## 4. Discussion

### 4.1. Principal findings

Nearly one in three Peruvian children suffer from anemia and it may be extensively explained by wealthiness, as it is distributed among the poorest. The departments with the highest magnitude of anemia-related inequalities in poor people were Ucayali, La Libertad, and Cusco. As well, the major contributing factors to the presence of inequalities were the mother’s education and region of origin. Additionally, it is necessary to consider other factors not evaluated that may explain the 18.15% residual contribution, such as, maternal anemia, early motherhood, high parity, short intergenesic period, or early umbilical cord clamping, among others (30). All in all, our study goes beyond describing the magnitude and distribution of inequalities in anemia among children, as it aimed to describe the contribution of the factors behind them.

### 4.2. Prevalence of anemia

The average prevalence of anemia in children under 12 years of age in Latin America is 28.56%, with variations that show a prevalence of 2.5% in Argentina and up to 67.59% in Haiti (10). A systematic review found that Peru is the country with the second highest prevalence of childhood anemia in Latin America, with a prevalence of 32.9% in children aged less than 5 years, higher than in our study (8). Likewise, in 2012, 20.7% of children had mild anemia, 11.8% had moderate anemia, and only 0.3% had severe anemia (31). Yet, we found that this figure improved in 2021, in which severe and moderate anemia decreased. Although the same population survey was used, this corresponded to the version conducted in 2012 and ours to the version conducted almost 10 years later. During this time, several government programs were launched to address childhood anemia, such as the “National Plan for the Reduction and Control of Maternal and Child Anemia and Chronic Childhood Malnutrition in Peru” (2017) and the “Multisectoral Plan to Fight Anemia” (2018), both of which aimed to reduce the prevalence of childhood anemia to 19% by 2021 (32). Unfortunately, according to our findings, we failed to achieve such goal.

The prevalence of anemia remains high despite the efforts made, posing a serious threat to the health of the population and the national economy. Even though the public budget for the “Articulated Nutritional Plan” (PAN, from the Spanish acronym) ascended to 2,511 million soles, only 355 million soles were estimated to be employed in the management of anemia in children. Of the total budget, 62.4% corresponded to diagnosis, 24.9% to follow-up, and 12.7% to treatment (33). Therefore, it is of utmost importance to allocate, administrate, and utilize efficiently the health resources to tackle anemia.

### 4.3. Inequalities in anemia

Anemia was distributed among the impoverished population, which poses further exposition to less access to a variety of food and, in turn, to low nutrient intake (12). Moreover, the travel time to health facilities is strongly correlated with the proportion of poverty and people with at least one unsatisfied basic need (34). These findings are in line with several studies from around the world. A multi-country study conducted in 45 low- and middle-income countries (LMICs), found that the socioeconomic inequalities of anemia persisted in the last years in roughly 80% of the LMICs evaluated, albeit nearly two-thirds of the countries showed a decrease in the prevalence (1). While most public policies are designed to improve the health status of the general population, the beneficiaries of these policies are more concentrated in sectors with higher SES (35). Despite the national antipoverty political agenda, which aims to improve child health and nutrition (36), significant inequalities in anemia remain in the country.

### 4.4. Contributing factors of inequality

Some sociodemographic characteristics may explain the inequalities of anemia in Peru. From 2012 to 2021, the percentage of children aged 6–59 months with anemia coming from rural areas and the lowest wealth quintile worsened (37). This suggests that the economic improvements achieved by our country did not necessarily have an impact on some health indicators, such as childhood anemia. Indeed, in Peru, *per capita* spending increased from 671 to 802 soles and monetary poverty went from 33.5 to 20.2% from 2009 to 2019, but these numbers worsened between 2019 and 2020, due to the COVID-19 pandemic (37, 38). This figure was shown mainly in the highlands where the largest number of poor people in our country are located (37).

The magnitude of inequality was heterogeneous between departments. In fact, Ucayali, La Libertad, and Cusco had the highest magnitude of pro-poor inequality. These disparities are likely influenced by the distribution of wealth and the distribution of the rural population in these departments. A recent report found that overall inequality between departments increased over the last decades (39). Moreover, according to our findings, being either from the highlands or the jungle, significantly contributed to the inequality of anemia. While the coast seems to be the most overall equitable natural region. There is no clear difference between the highlands and the jungle (39). Conversely, we found that the contribution of being from the highlands was higher compared to being from the jungle. People living in the highlands (the territory of the Andes) or the jungle (the rainforest between the Andean mountains and Brazil) are geographically disadvantaged, due to difficulties in access, and embrace other types of challenges related to the lack of understanding from healthcare professionals toward their cosmovision and conception of health, which widens the gap of inequalities (40).

Mother’s education, particularly higher education, contributed nearly a quarter to the overall inequality of anemia in children. As in the distribution of wealth between departments, the heterogeneity of higher education may also explain its contribution to the inequality of anemia (41). Enrollment in higher education increased from 23 to 31.2% between 2009 and 2019. Such increase was higher in the highlands by 8.7%, while on the coast was 7.6% and in the jungle was 7.8% (42). A recent cohort study found that children whose mothers had a low educational status were more prone to unhealthy nutritional habits (43). Poor maternal education would exert a negative impact beyond anemia. Indeed, a systematic review showed that maternal education is a strong predictor of child mortality (44). By improving maternal education, some within-family inequalities would be counteracted (45). All in all, improving mothers’ literacy skills is paramount to enhance health outcomes among children.

Although the relative contribution of health-related factors was not as large as others, together they have a significant impact on the distribution of inequality. Having incomplete vaccination coverage increases inequality. This topic is interesting because it is documented that anemia impacts negatively on adaptative immunity, making children prone to a reduced response to vaccines (46). Meanwhile, the use of antiparasitic drugs in the 6 months prior to the survey significantly reduced inequality. This is a low-cost but effective intervention that focuses on mitigating the unwanted consequences of unsafe water, poor hygiene, and lack of sanitation (47). In Peru, there is a higher prevalence of intestinal parasitosis in the poorest population (48), as well as precarious health facilities, despite improvements in recent years (49). A study assessing the association between water, sanitation, and hygiene conditions (WASH), and the prevalence of medical consultations for anemia found that these decreased by 0.22/1000 for each percentage point increase in the proportion of households with basic sanitation (50). It is important to mention that the prevalence of parasitosis in Peru is 64%, with a higher presence of protozoa on the coast and the highlands, and helminths in the jungle. Nevertheless, since 2017, the MINSA has started preventive campaigns that include the administration of antiparasitic drugs to people aged between 2 and 60 years old, in the framework of addressing anemia (51, 52).

### 4.5. Public health implications

Both the “National Plan for the Reduction and Control of Maternal and Child Anemia and Chronic Childhood Malnutrition in Peru” and the “Multisectoral Plan to Fight Anemia” propose improvements with emphasis on the poorest population (32). However, our results suggest that strategies should consider the factors contributing to inequality, which in turn influence the socioeconomic distribution of anemia prevalence, in order to have effective results. A study evaluating the effectiveness of government anemia programs in Madre de Dios found a lower probability of anemia in children exposed to anemia prevention programs compared to those not exposed (53). A meta-analysis showed that programs and health policies, especially those concentrated on nutritional interventions, reduced the prevalence of anemia from 45 to 25% in the LAC region (10). The most frequent interventions were food enrichment, micronutrient powders, and iron supplements delivery (2). However, it is reported that even with these efforts, the inequalities in low or very low SES remain due to other factors such as coverage, population compliance, monitoring, and quality in reporting results (2, 10).

### 4.6. Strengths and limitations

Our results must be interpreted considering their limitations. First, since our study was based on a secondary data analysis, it was not possible to include variables that would have been interesting to analyze, for instance, certain society- and environmental-related risk factors. However, only 6.76% of the records from children aged 6–59 months were dropped due to missing data. Second, we employed wealth status, which is based on assets, as a proxy measurement of SES owing to the lack a direct measure such as household income or consumption. Nevertheless, an asset-based approach is a suitable alternative for inequality analysis in the absence of a direct measure (54). On the other hand, our article also had several strengths. By adjusting sampling errors, it was possible to calculate representative estimates at the national and departmental levels. And, due to the large sample size, great statistical power is ensured. As well, the measurement method of the outcome variable was appropriate and accurate, particularly in large-scale community-based studies (55). Also, to the best of our knowledge, this is the first study carried out in children from the LAC region that untangles inequalities in anemia.

### 4.7. Conclusion

In conclusion, the national prevalence of anemia in Peruvian children was 29.47%. We found a pro-poor inequality of anemia in Peruvian children at the national level, but the magnitude of inequality was heterogeneous between departments. The determinants included in the model explained 81.85% of the overall socio-economic inequality in anemia among Peruvian children. Due to the large contribution of the region of origin, geographically designed policies are required to reduce disparities. As well, it is crucial to improve mothers´ education.

## Data availability statement

Publicly available datasets were analyzed in this study. This data can be found at: http://iinei.inei.gob.pe/microdatos/.

## Ethics statement

Ethical review and approval was not required for the study on human participants in accordance with the local legislation and institutional requirements. Written informed consent for participation was not provided by the participants’ legal guardians/next of kin because our study is based on a secondary data analysis of the Peruvian Demographic and Health Survey, which provided a written consents to the participants.

## Author contributions

AA-k-C, CM-G, and PR-V: conceptualization. AA-k-C: data curation. AA-k-C, CM-G, and LI-B: formal analysis. AA-k-C and LI-B: methodology. AA-k-C, CM-G, PR-V, KA, and PH-A: writing—original draft. AA-k-C, LI-B, KA, and VB-Z: writing—reviewing and editing. All authors contributed to the article and approved the submitted version.

## Conflict of interest

The authors declare that the research was conducted in the absence of any commercial or financial relationships that could be construed as a potential conflict of interest.

## Publisher’s note

All claims expressed in this article are solely those of the authors and do not necessarily represent those of their affiliated organizations, or those of the publisher, the editors and the reviewers. Any product that may be evaluated in this article, or claim that may be made by its manufacturer, is not guaranteed or endorsed by the publisher.

## Abbreviations

CC: Concentration curve
CI: Concentration index
DHS: Demographic and health survey
ECI: Erreygers concentration index
INEI: National institute of statistics and informatics
LAC: Latin American and Caribbean
SES: Socioeconomic Status
WHO: World Health Organization
