# Supplementary figures and images for: Inequalities in anemia among Peruvian children aged 6–59 months: A decomposition analysis
Source: Front Public Health. 2023 Mar 31;11:1068083. doi: 10.3389/fpubh.2023.1068083 (PMC10102391; doi:10.3389/fpubh.2023.1068083)

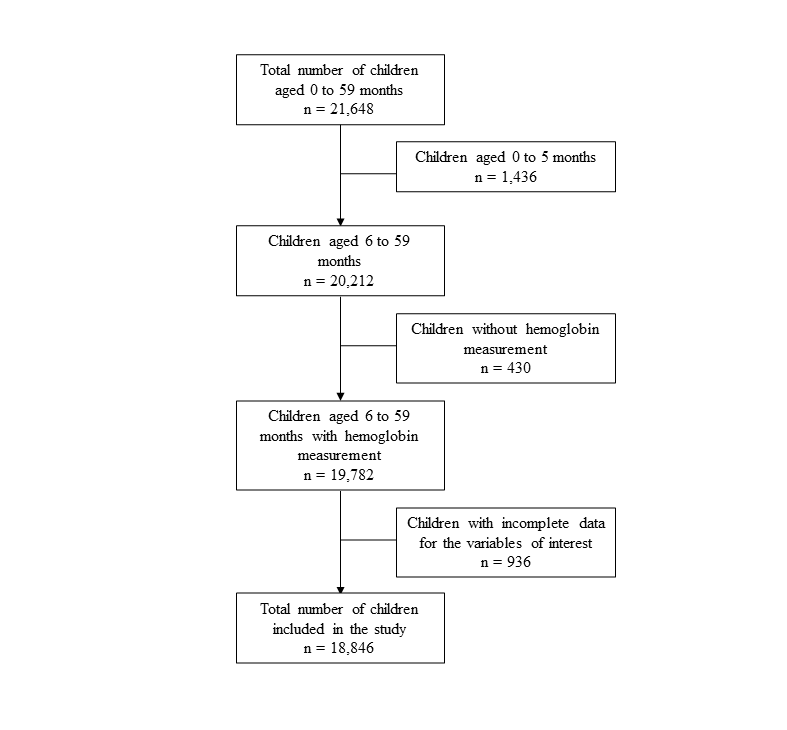

Supplement: Supplementary file 1 [file Image_1.TIF]

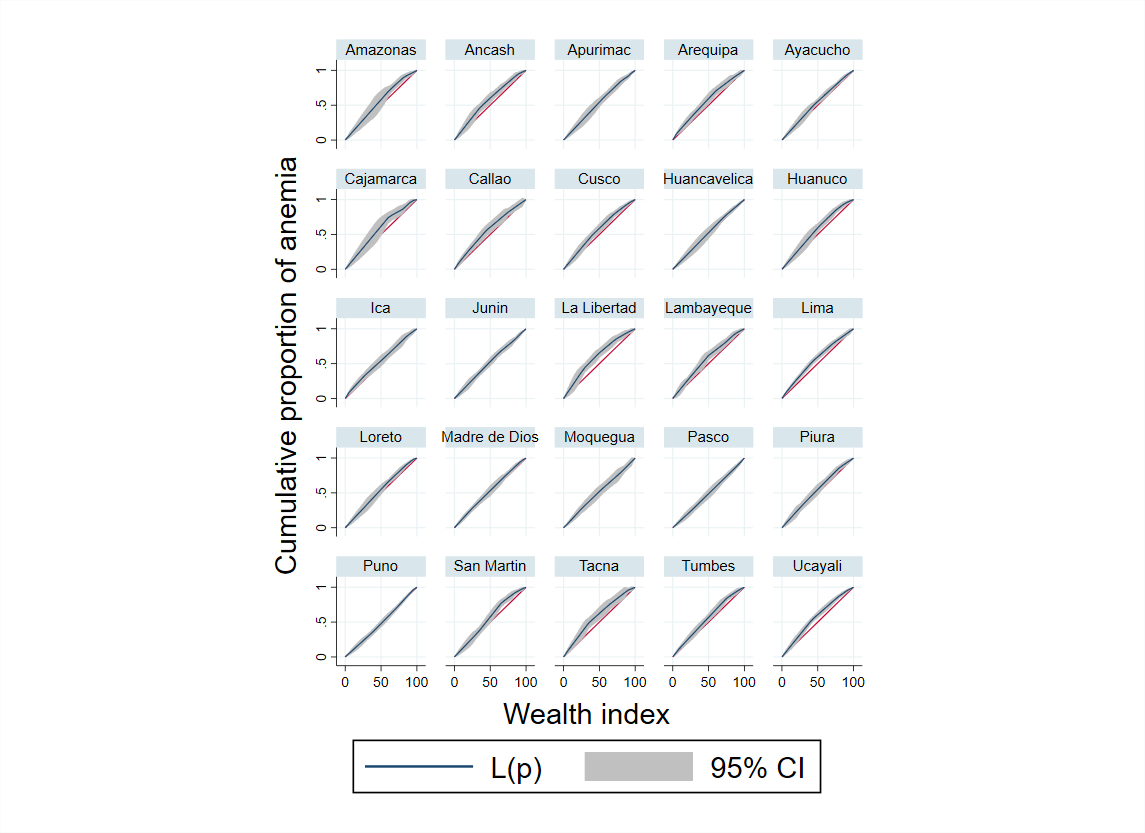

Supplement: Supplementary file 2 [file Image_2.TIF]
